# The Impact of the COVID-19 Pandemic on the Management of Mental Health Services for Hospitalized Patients in Sibiu County—Central Region, Romania

**DOI:** 10.3390/healthcare11091291

**Published:** 2023-04-30

**Authors:** Ciprian Băcilă, Laura Ștef, Mihaela Bucuță, Claudia Elena Anghel, Bogdan Neamțu, Adrian Boicean, Cosmin Mohor, Andreea Angela Ștețiu, Mihai Roman

**Affiliations:** 1 Dental and Nursing Medical Department, Faculty of Medicine, “Lucian Blaga” University of Sibiu, 550169 Sibiu, Romania; 2 Collective of Scientific Research in Neurosciences of the Clinical Psychiatry Hospital “Dr. Gheorghe Preda”, 550082 Sibiu, Romania; 3 Department of Psychology, Faculty of Social and Human Sciences, “Lucian Blaga” University of Sibiu, 550024 Sibiu, Romania; 4 Clinical Medical Department, Faculty of Medicine, The “Lucian Blaga” University Sibiu, 550169 Sibiu, Romania; 5 Research and Telemedicine Center in Pediatric Neurology, Pediatric Clinical Hospital Sibiu, 550166 Sibiu, Romania; 6 Preclinical Department, Faculty of Medicine, The “Lucian Blaga” University Sibiu, 550169 Sibiu, Romania; 7 Clinical Department of Surgery, Faculty of Medicine, The “Lucian Blaga” University Sibiu, 550169 Sibiu, Romania

**Keywords:** hospital management, COVID-19 pandemic, hospitalized patients, psychiatric hospital, mental health services

## Abstract

Introduction: The COVID-19 pandemic brought a burden and represented a challenge for the Romanian medical system. This study explored the consequences that COVID epidemiological measures had on the quality of the mental health care provided to hospitalized patients in a regional psychiatric hospital in Romania. Materials and methods: Both patient-level and hospital-level indicators were considered for this comparative retrospective study. On the one hand, we extracted patient-level indicators, such as sociodemographics, diagnosis, admission, and discharge dates for 7026 hospitalized patients (3701 women, average age = 55.14) from hospital records. On the other hand, for the hospital-level indicators, we included indicators referring to the aggregated concept of mental health services, such as case mix index, length of stay, bed occupancy rate and patients’ degree of satisfaction. Data extracted covered a period of two years (1 March 2019–28 February 2021) before and during the first year of the COVID-19 pandemic. Results: We found that, compared to the pre-pandemic period, the pandemic period was marked by a drastic decrease in hospitalized patient admissions, coupled with an increase in emergency-based admissions. Other management indicators, such as the case mix index, the number of cases contracted/performed, and the degree of patient satisfaction, decreased. In contrast, the average length of stay and bed occupancy rate increased. Conclusions: The COVID-19 pandemic, especially in the first year, raised multiple difficult issues for the management of psychiatric hospitals. It imposed an application of strict measures designed to face these new and unprecedented challenges. Our findings offer a detailed snapshot of the first year of the COVID-19 pandemic in terms of its impact on mental health services and suggest some future directions. Implications for hospital management are discussed.Keywords: hospital management; COVID-19 pandemic; hospitalized patients; psychiatric hospital; mental health services.

## 1. Introduction

Good hospital management depends on both offering high-quality health services and on the prompt and informed (preferably data-driven) and flexible allocation of resources. Once the COVID-19 pandemic was declared, health services started tobe severely impacted due to the epidemiological measures recommended by the World Health Organization (WHO). These measures aimed to limit the spread of infections and, implicitly, to reduce the pressure on medical health care providers by enforcing very strict restrictions [1,2,3]. Several studies [4,5,6,7,8,9] on the mental health of the population have been carried out and showed an increase in psychiatric manifestations, such as anxiety, depression, PTSD, and suicidal ideation [10], putting pressure on mental health care services. In Romania, the epidemiological measures also implemented in single specialty hospitals (psychiatry) [11] through the approved legislation [12,13] that has been changed considerably [14,15,16], continuously [17,18,19,20,21], not always coherent, and regulated in a particular way the health care activity in medical units [22,23] had consequences on structure and related functional circuits modification [24,25]. These changes had an impact, both on patients [26] and on employees of the medical system and were a challenge for mental health care/services management (Table S1).

In the aggregated concept of mental health services, we understand the way in which the management team provides support through easy access to medical care for psychiatric patients. This notion is defined by the following indices: the average duration of hospitalization that represents the sum of the number of days of hospitalization for all the resolved cases/number of resolved cases [27]; the complexity index of the cases which is calculated taking into account the total number of weighted cases (WC)/the total number of solved cases (SC) hospital [27]; the bed occupancy rate which is calculated using the bed utilization index × 100)/365 calendar days related to the reporting period [28]; the number of contracted/performed cases which is established through negotiation, depending on the number of performed cases and the way the qualitative indicators had been achieved in the previous period, taking into account the contracted beds, as well as the structural changes approved by the Romanian Ministry of Health or by the Directorate of Public Health [29]; the bed occupancy rate which is calculated using the bed utilization index × 100)/365 calendar days related to the reporting period [28,29,30], the degree of patient satisfaction as an indicator of the quality of the provided medical services [27,30].

The mental health care management provided during this pandemic was focused more on non-maleficence at the expense of beneficence, changing ordinary medical activity. Thus, the consequences of the COVID-19 measures in a psychiatric hospital were as the following [11]:

- Difficulties in limiting the transmission of SARS-CoV-2 virus infection, in providing complex medical care services to psychiatric patients infected, in respecting the rights of patients, the safety of medical staff and their involvement in the health care provided to patients.
- Reduced interaction time between patients and medical staff [31] with a negative impact on patients and medical staff due to the obligation to wear protective medical equipment [32,33]. Additionally, the contact was reduced by up to zero between patients [34,35] and isolation of the patients from their families [35].
- The impossibility of carrying out certain non-pharmacological programs [34,36] that require direct contact between patients and medical staff (e.g., suspension of occupational therapy and psychotherapy services [33], suspension of theater/melotherapy activities [37], related therapies that are all well known for their positive effects on these psychiatric medical health services [37,38].

The particularity of mental health care services in the Central Region of Romania, in Sibiu County, emerges from the existence of only one single specialty hospital that offers complex services with improved non-pharmacological programs and an outpatient department. In this unit, admissions are possible in case of emergency and by appointment, both voluntarily and involuntarily. A special case is an involuntary admission that is carried out on the recommendation of a psychiatrist, according to Romanian law no. 487/2002 [39], when patients have a severe intensity of psychiatric pathology and are not aware that they need proper treatment [40]. In that case, there is an increased risk for aggressive or violent behavior towards themselves or others. In addition to this hospital, there are also two psychiatric departments at other general hospitals, suitable only for admissions by appointment and without the possibility of involuntary admissions. The distribution of medical services is shown in Table A1 (Appendix A).

The primary objective of the present study is to determine if the COVID-19 epidemiological measures have modified the quality of the mental health care provided to hospitalized patients, therefore on mental health management. According to studies that have emphasized the increase of psychiatric pathologies in these times, we wanted to evaluate if the number of admissions and the length of stay in the psychiatric hospital have increased. As a secondary goal, for an objective viewpoint, we evaluated the management indicators, and for a subjective viewpoint, the patient satisfaction questionnaire (File S1) to understand how the COVID-19 pandemic has impacted mental health care.

## 2. Materials and Methods

The analysis of the impact of the COVID-19 pandemic and the epidemiological measures on the management of mental health services provided to hospitalized patients in Sibiu County was carried out by conducting a comparative retrospective study (Table S2) that included a sample of 7026 hospitalized patients, by emergency or appointment admissions, voluntary or involuntary, in Non-COVID or COVID departments.

### 2.1. Data

Data were extracted from hospital documents, such as patient records and observation charts provided by the statistics department, the involuntary admissions commission, and by the hospital’s public relations consultancy firm. We also extracted statistics regarding the weekly reported COVID-19 cases from public records (from 1 March 2020, and 28 February 2021). Data extraction was performed between March and June 2021.

Prior to data extraction, the ethics committee approval was obtained (1855/A2/15.02.2021) in accordance with the ethical principles and the declarations of Helsinki. Given the retrospective nature of the study and the fact that all data were anonymized, patient-informed consent for participation in this study was not required.

### 2.2. Sample

The patient-related data included a sample of 7026 adult psychiatric patients that were hospitalized between 1 March 2019–29 February 2020 (a pre-pandemic period) and 1 March 2020–28 February 2021 (the pandemic period). This particular time frame was chosen so as to include the onset of the pandemic-related lockdown, which was 1 March 2020 (in Romania), the year before (pre-pandemic) and the year after (pandemic) this time point of reference.

Both emergency and appointment admissions, as well as voluntary and involuntary admissions, were included. In the case of involuntary admissions, all legal requirements under Romanian laws (Law 487/2002) were followed [30]. For the pandemic period, data included adult patients from both COVID-19 and Non-COVID-19 departments. An exclusion criterion was the chronic type of hospitalizations (under ICD-10 classification criteria), such as patients under palliative care (i.e., that cannot be integrated into society due to the severity of their psychiatric condition).

### 2.3. Variables and Indicators

#### 2.3.1. Patient-Related Variables/Indicators

Data extracted from hospital records included socio-demographic characteristics, specifically, patient gender, age, area of residence (rural/urban) and occupation. Patients’ main psychiatric pathology (according to ICD-10, codes F00-F99) was coded under more general diagnostic categories as follows: mood disorders, psychotic disorders, personality disorders, addictions (e.g., ethanol, cannabis), cognitive decline, mental retardation, and other pathologies (e.g., neurosis).

For each patient, we also extracted admission and discharge dates and computed the number of hospitalized days. Based on their admission date and the chosen time point of reference (1 March 2020), we classified patients as admitted either during the pre-pandemic period or the pandemic period.

#### 2.3.2. Hospital-Level Indicators

The indicators chosen at the hospital level were: (1) the average duration of hospitalization for resolved cases [27], (2) the case mix index [27], (3) the bed occupancy rate [28], (4) the ratio between contracted and performed cases [29], and (5) the patient’s satisfaction [30]. Table 1 provides details regarding how each indicator was computed, the scale of measurement (e.g., percentage), the temporal granularity of the available data (monthly or by a period of interest) and the data source.

### 2.4. Analytical Approach

We reported descriptive statistics (frequencies, central tendency and dispersion indicators), as well as inferential tests, where appropriate. For descriptive statistics expressed as percentages, we additionally computed the percentage of change between the pre-pandemic and pandemic indicators by subtracting the pre-pandemic-related percentage score from the pandemic percentage score, then dividing the difference by the pre-pandemic percentage score and multiplying it by 100 (to indicate percentage). For inferential statistics, we ran chi-square tests of independence for cross-tabulations and reported Cramer’s *V* as an effect size indicator. Group (time period) comparisons were made using either t-tests or analysis of variance, both with Welch correction for unequal variances. For each analysis, we report appropriate unbiased effect size estimates (i.e., Hedge’s *g*, partial omega squared) and their 95% confidence intervals.

All patient-related data were analyzed and plotted using R programming language (version 4.2.3) [41]. R code and datasets are available upon request.

## 3. Results

### 3.1. Patient-Level Indicators

#### 3.1.1. Overall Sample Characteristics

Table 2 shows the full sample’s demographic characteristics (N = 7026). There were slightly more female than male patients in this sample (5.4% more females). The average patient age was around 55 years old, and more than 60% resided in urban areas. Additionally, the majority of patients were either retired or without an occupation.

#### 3.1.2. Patient Admission Frequency

**Figure 1:**
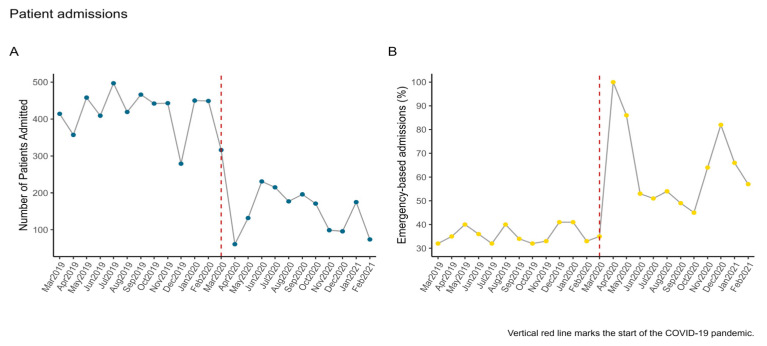
Panel (**A**)—Evolution of patient admissions throughout the time frame of interestand Panel (**B**)—Time series of emergency-based admissions.

Figure 1A illustrates that the decrease in patient admissions occurred right after lockdown measures were enforced by authorities and that the level of admissions remained relatively low throughout the remaining time frame of interest. We used a one-sample chi-square test to compare patient admission frequencies between the pre-pandemic and pandemic periods under the null hypothesis that expected frequencies would have the same probability. Findings showed that there was a statistically significant drop in cases in the pandemic period relative to the pre-pandemic period, *χ^2^* (1) = 17.09, *p* < 0.001.

#### 3.1.3. Emergency-Based Admissions

Figure 1B shows that during the pandemic period, emergency-based admissions drastically increased (reaching 100% in April 2020) at the start of the pandemic and again in December 2020 (the second wave of the pandemic). Overall, emergency-based admissions exceeded 50% after the onset of the pandemic, compared to the pre-pandemic period, when this type of admission remained around 40% or lower.

#### 3.1.4. Involuntary Admissions

**Figure 2:**
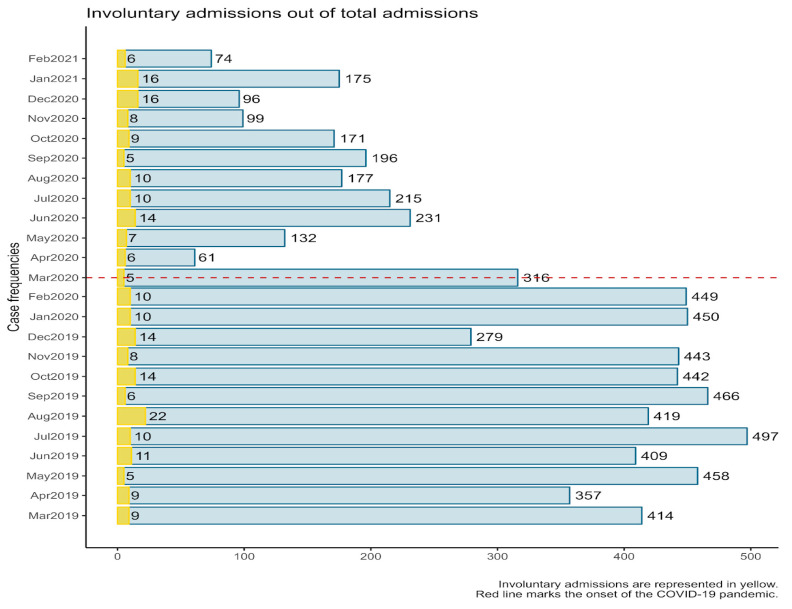
Monthly involuntary admissions out of total admissions for the time frame of reference.

Figure 2 shows while the overall number of monthly cases before, compared to after the onset of the COVID-19 pandemic (1 March 2020; marked with a red dotted line) was significantly reduced (see Section 3.1.2), the number of involuntary admissions remained fairly constant throughout the two periods of interest. A one-sample chi-square test to compare involuntary patient admission total frequencies in the pre-pandemic and pandemic periods, under the null hypothesis that expected frequencies would have the same probability, revealed no statistically significant differences, *χ^2^*(1) = 1.07, *p* = 302.

### 3.2. Comparison between Pre-Pandemic and Pandemic Periods

#### 3.2.1. Sociodemographics

We examined whether there were significant differences in the sociodemographic characteristics of the pre-pandemic sub-sample compared to the pandemic sub-sample. There were no significant differences in the gender distribution of patients depending on time period, with 47% male patients admitted in the pre-pandemic period and 48.3% male patients admitted in the pandemic period), *χ^2^*(1) = 0.89, *p* = 346.

There was a small but significant difference between pre-pandemic and pandemic samples in terms of age. The average age of patients admitted during the pre-pandemic period was higher, M = 55.99, SD = 14.89, compared to the average age of patients admitted during the pandemic period, M = 52.67, SD = 15.32, *t*_Welch_(3390.4) = 7.43, *p* < 0.001, Hedges *g* = 0.20, 95% CI[0.15, 0.25].

**Figure 3:**
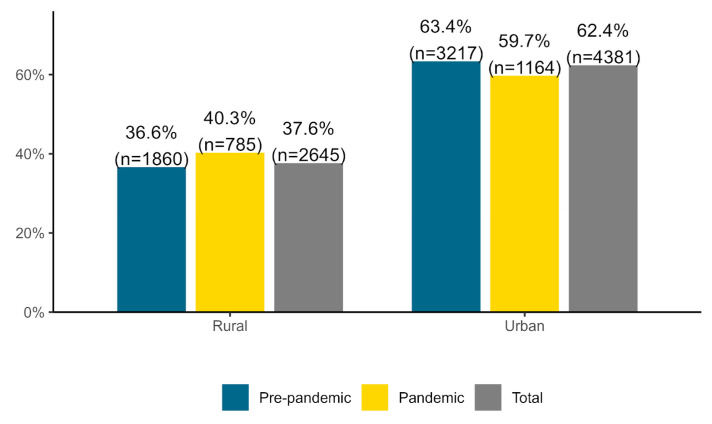
Total pre-pandemic and pandemic percentages for patient residence.

Compared to the pre-pandemic period, the percentage of patients residing in rural areas increased by 10.11% in the pandemic period (40.3%) relative to the pre-pandemic period (36.6%) (see Figure 3). However, the percentage of patients from urban areas decreased by 5.84% in the pandemic (59.7%) relative to the pre-pandemic period (63.4%). These changes were statistically significant, *χ^2^*(1) = 7.95, *p* = 0.005, Cramer’s *V* = 0.03.

**Figure 4:**
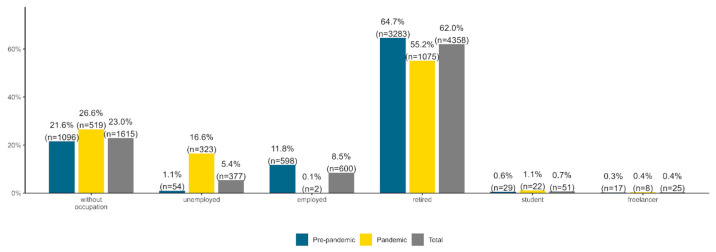
Total, pre-pandemic and pandemic percentages for patient occupation.

In the case of occupation categories, relative percentages varied significantly depending on time period, *χ^2^*(1) = 898.499, *p*< 0.001, Cramer’s *V* = 0.36 (see Figure 4). We computed the relative percent changes from the pre-pandemic period to the pandemic period. The percentage of patients that were retired or employed decreased by 14.68% and 99.15%, respectively, in the pandemic, compared to the pre-pandemic period. Conversely, compared to the pre-pandemic period, relative percentages of patients from all the other occupation categories increased in the pandemic period by 18.8% (without occupation), 1409.09% (unemployed), 83.33% (student), and 33.33% (freelancer).

#### 3.2.2. Diagnoses

**Figure 5:**
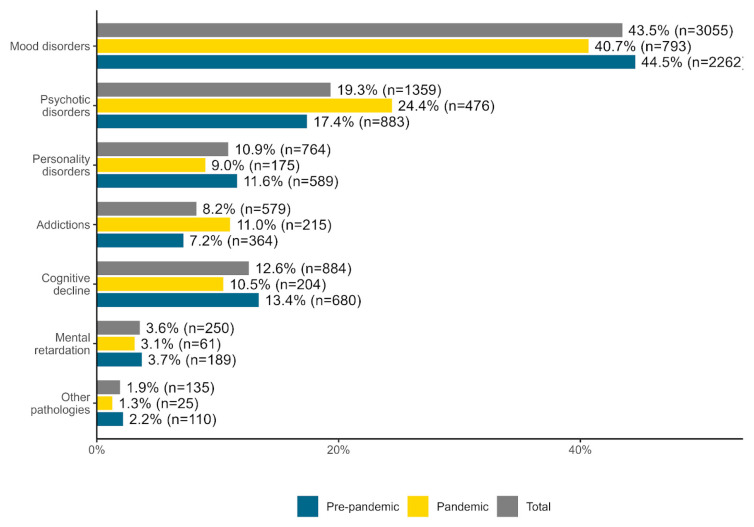
Total, pre-pandemic and pandemic periods percentages for each diagnosis category.

Depending on the time period, diagnostic categories differed slightly but significantly, *χ*^2^ (6) = 91.932, Cramer’s *V* = 0.114, *p* = 0.001. On the one hand, compared to the pre-pandemic period, during the pandemic period, there was a decrease in the relative proportion of admitted patients diagnosed with a mood disorder (8.54%), with personality disorder (22.41%), with cognitive decline (21.64%), mental retardation (16.22%) and other disorders (40.91%; e.g., neurosis). On the other hand, there was an increase in the relative proportion of patients admitted for psychotic disorders (40.23%) and diagnosed addictions (52.78%) (see Figure 5).

#### 3.2.3. Average Length of Stay

For each patient, we computed the number of days they were hospitalized (between the admission date and discharge date). The distribution of this variable was positively skewed, meaning that there were extreme cases in which patients spent more than 200 days admitted. To address this issue, we computed the interquartile range (IQR; the difference between the values of the 75th and the 25th percentiles) and then calculated the lower and upper bounds by multiplying the IQR with 1.5 and subtracting it from/adding it to the value of the 25th/75th percentile.

Across patients and timepoints, the median number of days was 12 (M = 15.01, SD = 14.32). The 25th percentile value was 8, and the 75th percentile value was 17, giving an IQR of 9. No exclusions were made based on the lower bound, which was below 0. However, the upper bound was 30.5; therefore, all patients that exceeded 30.5 total days of admittance were removed from this analysis. There were 640 patients that exceeded 30.5 total days of admittance; these patients were excluded from further analyses that involved this variable. Within this sub-sample, the average length of stay was 47.77 (SD = 26.08, IQR = 16, range = [31,356]).

The pandemic had a small but statistically significant impact on the average length of stay. Specifically, the average number of days spent admitted increased during the first year of the pandemic (n = 1703), M = 12.72, SD = 7.05, relative to the previous, pre-pandemic (n = 4683) year, M = 11.36, SD = 5.92, *t*_Welch_(2623.82) = −7.08, *p*<0.001, Hedges’ *g* = −0.21, 95% CI[−0.27, −0.15].

#### 3.2.4. Length of Stay and Diagnosis

**Figure 6:**
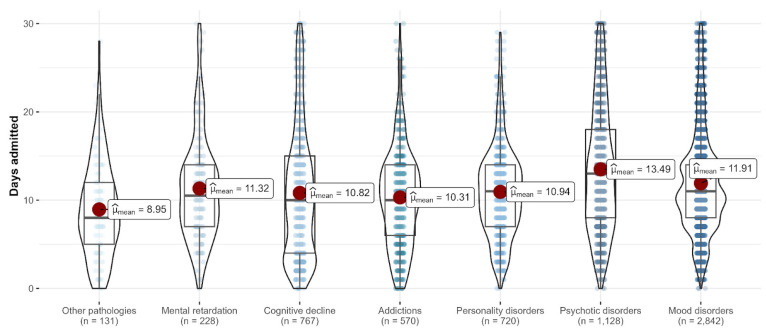
The average number of days admitted depends on the diagnosis.

Additionally, we conducted a one-way between-subjects analysis of variance to compare the average length of stay by diagnostic category. There was a large significant main effect of the diagnostic category, F_Welch_(6, 1019.75) = 28.09, *p*< 0.001, ω^2^ = 0.14, 95% CI[0.10, 1.00]. Figure 6 shows that patients with psychotic disorders had the longest average admittance duration (approx. 14 days), followed by patients diagnosed with mood disorders (approx. 12 days) and with mental retardation (approx. 11 days). Games-Howell posthoc tests (appropriate for unequal variances between groups), using the Holm method to adjust *p*-values, revealed all pairwise comparisons as significant at *p* < 0.05, except for pairwise comparisons between addictions, personality disorders, cognitive decline and mental retardation, as well as between mental retardation and mood disorders and between cognitive decline and other pathologies.

#### 3.2.5. Patient Hospital Admissions and COVID-19 County-Level Infection Rates

**Figure 7:**
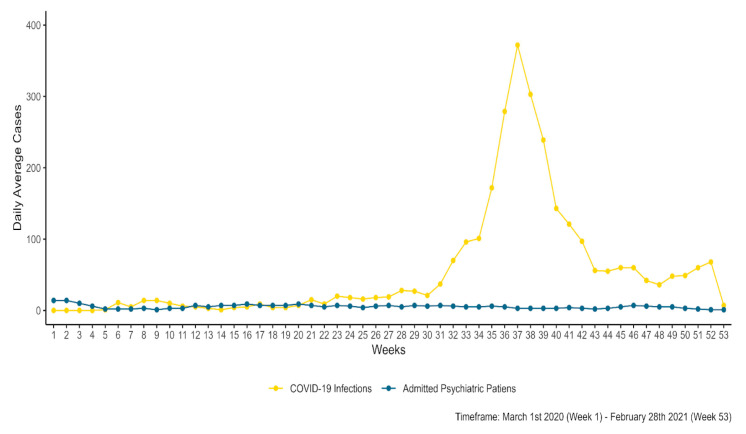
Evolution of daily average patient admissions and county-level COVID-19 infections.

County-level COVID-19 data regarding the number of infections per week were extracted from public records and were matched with the time frame of our study (only for the pandemic sub-sample). We computed the daily average infections for each week and then plotted them against the daily average number of patients admitted at the psychiatric hospital for each of the 53 weeks included in the time frame of interest. Figure 7 shows that while COVID-19 daily average infections fluctuated, daily average admissions remained constant during the analyzed period.

### 3.3. Hospital-Level Indicators

#### 3.3.1. Case Mix Index

**Figure 8:**
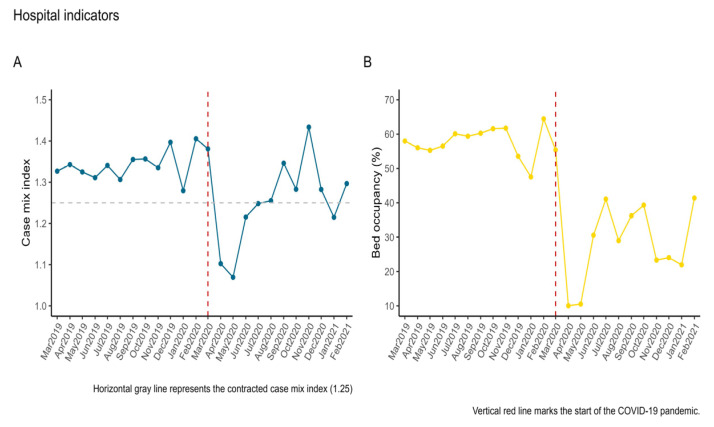
Panel (**A**)—Time series of case mix index and Panel (**B**)—Time series of bed occupancy rate.

The case mix index contracted annually by the psychiatric hospital is 1.25. Before the onset of the pandemic, the performed case mix index was relatively constant and above the contracted value. However, after the onset of the pandemic, the case mix index of performed cases dropped during the first four months (March–June 2020) and slowly recovered and even exceeded the contracted values until December 2020 (see Figure 8A).

#### 3.3.2. Bed Occupancy Rate

The optimal bed occupancy rateat the psychiatric hospital for acute cases is 79.45%.

Figure 8B illustrates that compared to the pre-pandemic period, where the average occupancy rate was 57.82%, at the onset of the pandemic, the bed occupancy rate dropped to a value as low as 10.03% in the first month of the pandemic, and increased only after three months, but remained under 50% for the remaining time frame of interest.

#### 3.3.3. Performed/Contracted Cases

The total number of contracted cases at the single specialty psychiatric hospital during the pre-pandemic period (March 2020–February 2021) was 5461. However, during the pandemic period, due to the measures imposed, there were no cases contracted. Implicitly, the average number of performedcases in the pre-pandemic period was higher (482.66) relative to the pandemic period (190.4). The average value of performed cases decreased significantly during the pandemic period, with very low values recorded in April (58), May (60), November (125), December (98), and January (140).

#### 3.3.4. Patient Satisfaction

The hospitalized patients’ perceptions of received medical services were assessed with a satisfaction evaluation questionnaire that contained multiple aspects covering both the medical act and several aspects regarding how the patients interacted with the staff and hospital environment/facilities. Based on the aggregated percentages (across patients and by time period), we computed the percentage of change in satisfaction scores for each index between the pre-pandemic and pandemic periods. Overall, patient satisfaction decreased on all indices. The largest changes (that can be directly attributed to the pandemic) referred to hospital recreational resources/activities and medical personnel identity. During the first year of the pandemic period, patients at the hospital in question reported being less satisfied with the ward environment, physician-patient communication, and nurses’ quality of care (see Table 3).

## 4. Discussion

We found that the mental health services provided to hospitalized psychiatric patients were influenced by the COVID-19 pandemic regarding the admission number, urban/rural areas, the patient’s diagnosis, average length of stay, case mix index, bed occupancy rate, performed/contracted and patients’ satisfaction. We are discussing further each of these results.

### 4.1. Number of Admissions

#### 4.1.1. Patient Admission Frequency

Although numerous studies have highlighted that the COVID-19 pandemic had unfavorable consequences on the evolution of patients with psychiatric pathology [4,5,6], there was a decrease in the number of admissions in psychiatric facilities worldwide [42,43,44,45,46,47]. The results of this study show that the COVID-19 pandemic influencedthe number of admissions by decreasing them, similar to those obtained by Fasshauer J.M. et al. (2021) [47], who, in their study, had decreased admissions by 60% [47]. A possible explanation for this fact can be the reduced number of beds [48], by 50% of the hospitalization capacity in a room or by allocating 23 beds to the COVID-19 department, measures regulated by the specific legislation [33,48], and in order to limit the spread of the SARS-CoV-2 virus through social distancing within. The fear of contamination with the new virus and misinformation from the mass media [11,49] could have been factors that determined the decreased number of admissions. According to the World Health Organization [50], the infodemic generated by the media represented a new epidemic of false information affecting the population concomitantly with the COVID-19 pandemic [51]. In a study, Brenner S.J. et al. 2020 demonstrated that important public figures were responsible for the spread of misinformation, creating an even greater fearin the observance and application of the epidemiological measures, of the perception of the medical system and of the services offered by it [52].

#### 4.1.2. Patient Hospital Admissions and COVID-19 County-Level Infection Rates

The fear of contamination with the SARS-CoV2 virus being again a determining factor in accessing medical services [11,49] influenced the presence of admitted patients to the psychiatric hospital. As a particular aspect of this pandemic period, in November 2020, Sibiu County had the highest rate of COVID-19 infections [53], accumulated at more than 14 days, in Romania (7.78‰); this influenced the admissions by appointment in December.

#### 4.1.3. Emergency-Based Admissions

During the pandemic, the increased extended period of time that patients accessed mental health services within the psychiatric hospital led to the expansion of emergency-based admissions, thus, the decrease in appointment admissions [40]. This decrease can be explained by the uncertain epidemiological history of the patients and the fear of psychiatrists admitting them due to the possible risk of increasing the number of infections with COVID-19 in the medical unit [44].

#### 4.1.4. Involuntary Admissions

Although the number of emergency admissions increased during the pandemic period, the involuntary admissions, which were carried out at the recommendation of the psychiatrist, according toLaw no. 487/2002 [39], when the psychiatric pathology of the patients was of severe intensity, and they were not aware of the need for appropriate specialized treatment [40], remained relatively constant. These results are in agreement with others reported by Clerici et al. (2020) [43] and in disagreement with those reported by Bonello et al. (2021) [54], who showed that involuntary admissions between the pre-pandemic and pandemic periods studied by them had statistically significant differences.

### 4.2. Sociodemographics

From the sociodemographic factors, low-income status is the one that had a significant influence during the pandemic on hospitalized admissions. The increased percentage of patients residing in rural areas during the pandemic period can be explained by the difficult access of these patients to outpatient medical services, on-site and through telemedicine service.

### 4.3. Diagnoses

Numerous studies carried out during this pandemic period [4,5,6] showed that patients with psychiatric pathology had an unfavorable evolution. Gobbi et al. (2020) carried out a study that showed that half of the evaluated patients with known psychiatric pathology had accentuated psychiatric symptoms [55]. The consequences of the pandemic measures, like anxiety and social isolation, were reported as determining factors in the decompensation of affective pathology [56,57].

The results of our study about mood disorders were in disagreement with the findings reported by the authors mentioned above, indicating that there was a decrease of 8,54% in the admissions. Evaluating the pandemic consequences on the mental health of COVID-19 hospitalized patients, Sahan et al., 2021 [58] found that 4 out of 10 patients developed depression and 3 out of 10 patients had symptoms of anxiety [58]; Liu et al., 2020 [59] reported a correlation between the increased risk of anxiety, the corticosteroid treatment [59] and the degree of SARS-CoV-2 severity infection. Additionally, Turane et al. (2021) [60] found that 10% of the admitted patients required psychiatric consultation during hospitalization [60], 19% for depression and 9% for suicidal ideation [60,61]. In addition to this, in the COVID department of the Psychiatric Hospital, out of the total number of admissions, we found that 33.41% of patients were diagnosed with a mood disorder, dominated by suicidal ideation and autolytic attempts [6], with the predominance of admissions in the female sex [62]. 40% of these admissions were patients without psychiatric history, with the onset of autolytic ideation in hospitalization conditions, in the red wards from the general hospitals or from departments of infectious diseases that treated exclusively positive COVID-19 patients.

The impact of the COVID-19 pandemic on the patients’mental health [6] could also be emphasized by the increased number of deaths due to suicide in the red wards located within general hospitals [63]. We can only make assumptions and advance the idea that the isolation measures, the stigma of being a positive patient, the fear of the disease, of new psychiatric pathologies, the economic effects that appeared during this period, such as unemployment [64,65], could have been the triggering factors of increased suicides. More specific research, like the psychological autopsy performed on patients who have died by suicide, would have had a crucial role in understanding the context in which this suicide occurred [66].

We also found a decrease in admissions for patients diagnosed with personality disorders, cognitive decline, and mental retardation. This can most likely be due to the fact that the families delayed the hospitalization of these patients due to the fear of contamination and especially to the fact that, during this period, the rate of illnesses and deaths in elderly patients was high [34,61,67]. 

On the other hand, we found admissions for patients diagnosed with psychosis increased by 40.23% during the pandemic. These results can be interpreted both as a consequence of the epidemiological measures and of the delay in accessing specialized medical services due to the fear of contamination [11,49], the social isolation triggered by incorrect information from the mass media [51,52] or the lack of a social support network [68]. Our results are not consistent with the results of the studies conducted by Bonello et al.(2021) [54] and Clerici et al. (2020) [44].

Taylor et al. (2020) highlighted that alcohol consumption was used as a substitute for specialized treatment in the mechanism of stress adaptation [69]. We found an increase of 52.78% in admissions of patients diagnosed with addiction. This fact can be correlated with social distancing measures, emphasizing the feeling of loneliness and emotional distress, as well as the low accessibility for procuring alcohol, with relapses that can influence the decompensation of these patients [70,71].

### 4.4. Length of Stay

#### 4.4.1. Average Length of Stay

Although the number of admissions during the pandemic period decreased, the average length of stay increased from 11.36 days in the pre-pandemic period to 12.72 days inthe pandemic period, the results being in accordance with the studies carried out in Italy [44] and Germany [47]. This can be interpreted due to the change of the admitted procedure within the psychiatric hospital, of the uncertainty related to the SARS-CoV-2 virus incubation or the reference RT-PCR (Polymerase Chain Reaction) tests [72] for the specific diagnosis of COVID-19, therefore generating implicitly additional costs for the psychiatric hospital. Some other situations, like COVID-19 comorbidities, the difficulty of socio-familial reintegration due to the lock-down or the increase in the positivity rate with the SARS-CoV-2 virus among the community where the family members were isolated or hospitalized in the wards of infectious diseases, could also determine the increase in the average length of stay [44].

#### 4.4.2. Length of Stay and Diagnosis

Although we determined the average length of stay during the pandemic period, we found that the longest average admittance *was* on psychotic patients with 14 days. This can be explained due to the severity of the symptomatology, the admission avoidance, and, possibly, because of the slow answer to the antipsychotic treatment.

### 4.5. Hospital-Level Indicators

#### 4.5.1. Case Mix Index

Despite the increase in the number of somatic complications due to the COVID-19 pandemic multiple medical challenges, the case mix index during the pandemic period decreased, though Sibiu County had the highest incidence in Romania of SARS-CoV-2 virus infections.

The increased attention of the medical staff in providing specialized medical care to patients, neglecting the logistics aspects of the files, being a very demanding period for the medical staff, could have led to the appearance of burnout within this professional category [73], manifested through repercussions on their professional activity, some doctors becoming more superficial, especially in completing medical documents [73]. During this period, the finance of the hospital’s case mix index was no longer considered, as it was in the pre-pandemic period, because of the additional expenses generated by the COVID-19 pandemic. These units wouldn’t have been able to continue their activity with important consequences for the quality of the medical act.

#### 4.5.2. Bed Occupancy Rate

Thus, looking at the results obtained during the pandemic period in the months of April, May, June, November, December, and January, one notices that there is a causal relationship between the rate of use of beds and the number of performed cases, values that are found a lot below the optimum average, a fact that can also be explained by the fear of contamination with the new virus, as well as by misinformation from the mass media [11,49].

#### 4.5.3. Number of Cases Contracted/Performed

The number of cases contracted/performed during the pandemic decreased. Thus, it was a need to change the financial support provided by the National Health Insurance House (NHIH). If during the pre-pandemic period, the financing was carried out according to the contracted/ performed cases, by the NHIH and the National School of Public Health, Management and Improvement (NSPHMI), during the pandemic period, there were unlimited funds that covered all the expenses, in order to ensure the continuity of the quality of medical services and the salaries payment of the employed staff in the hospitals.

#### 4.5.4. Patient Satisfaction

The significant decrease during the pandemic period confirms that, although epidemiological measures were respected, these had a negative impact on the quality of the medical services provided to patients. The determining factors in the patients’ satisfaction decline were: a decrease in interaction and communication between patients and medical staff (due to the wearing of protective equipment) [3,31,33], the interaction between patients [35,74], restriction until the cessation of related services [33,34,37], services with a purpose in creating a relaxing environment and a therapeutic role [37,38]. The isolation from their families [35] for a longer period of time, as well as the uncertainty of the probable date of discharge, was also a difficult situation to handle.

### 4.6. Limitations

Due to the fact that in Sibiu County, there is only one single specialty hospital that can offer complex mental health services, the sample of the study included data only from hospitalized patients from this institution. For a better comprehensive understanding of the mental health management system in the central region, Romania will be needed further studies.

Another limitation of the study was in our sample the fact that we did not analyze the recurrence of the patients’ admissions which is an important factor to evaluate in further studies in order to have a wider view of the pathologies’ evolution.

## 5. Conclusions

During the pandemic period, the number of admissions from the psychiatric hospital of Sibiu County, Central Region, Romania, decreased along with the increased average length of stay, having repercussions on the index case mix, which was below the value of the contracted one, on the average value of performed cases, on the degree of patient satisfaction regarding the quality of the medical services. In addition to these findings, as far as the diagnosis at admission is concerned, the number of hospitalized patients with psychoses and addiction increased; furthermore, regarding affective pathology, much to our surprise, decreased. The results of this study align with other similar ones and reinforce the need to create national programs in such crisis situations.

Carrying out a comparative retrospective study gave us opportunities to have a detailed snapshot of the first year of the COVID-19 pandemic in terms of its impact on mental health services provided to hospitalized patients, requiring different approaches and adjustments for maintaining the quality of the medical act [11,75]. It can be interpreted as a hypothetical exercise for a possible large-scale disaster [75]. Considering the impact of the pandemic on the management of mental health services, the recommendation for professionals in the field would be to create certain mental health services and screening programs, for instance, in order to detect autolytic ideation that appeared as a consequence of critical situations or in the professional field, in order to prevent the burn-out in the medical system.

### Future Directions

Together with the measures imposed by the COVID-19 pandemic come the structures that can be used in the future as a solution to improve mental health management and the quality of the medical services provided within psychiatric hospitals, establishing new departments for treating psychiatric patients with somatic comorbidities (infectious diseases, such as those caused by Clostridium difficile, HIV, TB).

Whether we discuss natural or social conditions (war, economic crises), it is important to have mental health programs (emergency interventions) that can be implemented at any moment, with a higher degree of accessibility and great adaptability in the provision of medical care.

## Figures and Tables

**Table 1:** Hospital-level indicators are included in the aggregated concept of mental health.

Indicator	Metric	Formula/Items	Temporal Unit	Data Source
Average length of stay for solved cases	days (average)	Sum of number of days for solved cases divided by number of solved cases	Monthly	hospital and public records
Case mix index ^a^	Ratio	Total frequency of weighted cases divided by the frequency of solved cases	Monthly	hospital records
Bed occupancy rate	percentage	Total number of hospitalized patient days ^b^ by period/(available beds × 365 days) × 100	Monthly	hospital records
Contracted/performed cases ^c^	frequency/ ratio	Not applicable	Period	hospital records
Patient satisfaction	percentage	Informing patients Quality of care Medication administration Physician-patient communication Medical personnel identity Hospital recreation resources/activities Overall service quality	Period	hospital public relations provider

Note: ^a^ The case mix index refers to the level of complexity of cases. Weights are applied according to the diagnostic-related group (DRG), which refers to an assigned average value based on the resources needed to treat patients with a given (psychiatric) diagnosis. ^b^ Hospitalized patient days refers to the actual number of beds occupied by patients in a given period. ^c^ The number of contracted/performed cases is established through negotiation, depending on the number of performed cases and the way the qualitative indicators had been achieved in the previous period. It takes into account the contracted beds, as well as the structural changes approved by national authorities (i.e., Ministry of Health, Public Health Department).

**Table 2:** Sociodemographic characteristics.

Variable	Details	Values Frequency (%) or M (SD)	Distribution
Gender	1. Male 2. Female	3325 (47.3%) 3701 (52.7%)	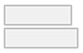
Age	Minimum = 18 Median = 56 Maximum = 97 IQR = 19	55.1 (15.1)	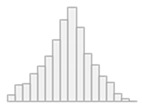
Residence	1. Rural 2. Urban	2645 (37.6%) 4381 (62.4%)	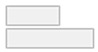
Occupation	1. without occupation 2. unemployed 3. employed 4. retired 5. student 6. Freelancer	1615 (23.0%) 377 (5.4%) 600 (8.5%) 4358 (62.0%) 51 (0.7%)25 (0.4%)	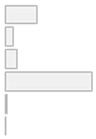

**Table 3:** Patient satisfaction indices in the pre-pandemic and pandemic periods.

Index	Type	Period		% Change (Decrease)
		Pre-pandemic	Pandemic	
Informing patients	Rights, Obligations, Regulations	98%	90%	8.16%
	Medication side-effects	90%	87%	3.33%
	Diagnosis	90%	85%	5.56%
	Approximate discharge date	91%	74%	18.68%
Quality of care	Doctors	82%	80%	2.44%
	Nurses	91%	70%	23.08%
	Orderlies	80%	72%	10%
Medication administration	Oral	87%	85%	2.30%
	Infusion	87%	86%	1.15%
Physician-patient communication		90%	60%	33.33%
Medical personnel identity		93%	52%	44.09%
Ward environment		90%	68%	24.44%
Hospital recreation resources/activities		90%	30%	66.67%
Overall service quality		90%	82%	8.89%

Note. The percent change (decrease) was computed as the percent difference between the intra-pandemic percentage score and the pre-pandemic percentage score, divided by the pre-pandemic score and multiplied by 100 (for percentage).

## Data Availability

The data can be provided upon reasonable request.

## Supplementary Materials

The following supporting information can be downloaded at: https://www.mdpi.com/article/10.3390/healthcare11091291/s1, Table S1: Impact of the challenges illustrated by the study’s results on the measures taken; Table S2: STROBE Statement—Checklist of items that should be included in reports of cohort studies [76]; File S1: The English version of the Satisfaction Questionnaire.

## Appendix A

**Table A1:** The situation of mental health services in Sibiu County- shows the breakdown of psychiatric services, the number of beds, as well as outpatient services, with the related distribution of psychiatric doctors.

Cities	Population	Monospeciality Hospitals	Department of Psychiatry in Hospitals	Outpatient Clinics Integrated within Hospitals	Mental Health Centers	Offices under Contract with CNAS	No. of Psychiatrists	No. of Beds
Sibiu	155,000	1	1	5	1	12	33	453+(10)
Medias	51,715	-	-	1	-	3	6	
Cisnadie	14,665	-	-	1	-	1	2	-
Agnita	10,242	-	1	1	**-**	-	1	(8)
Ocna Sibiului	4210	-	-	-	**-**	1	1	
