# Supplementary material for: The Impact of the COVID-19 Pandemic on the Management of Mental Health Services for Hospitalized Patients in Sibiu County—Central Region, Romania
Source: Healthcare (Basel). 2023 Apr 30;11(9):1291. doi: 10.3390/healthcare11091291 (PMC10178149; doi:10.3390/healthcare11091291)
Supplement: Supplementary file 1 [file healthcare-11-01291-s001.zip › File S1.pdf]

SUPPLEMENTARY MATERIAL

File S1

PATIENT/FAMILY MEMBER SATISFACTION EVALUATION QUESTIONNAIRE
for continuous hospitalization departments

(Translated from Romanian)

Dear Patient/Family Member,

In order to assess the care and medical services you have received at the "Gheorghe Preda" Psychiatry Hospital in Sibiu and to continuously improve their quality, we kindly ask you to answer the questions in the questionnaire below. After completing it, please deposit it in the specially designated spaces at the level of the department or compartment that provided you with these services.
In the case of minors or patients without discernment (temporary or constant), the legal representative of the patient will complete the questionnaire.
The hospital assures you of the confidentiality of your answers. The information obtained will not be associated with you and will not affect the quality of care and your relationship with the attending physician.
Please answer the questions by checking the option that best describes your situation.
Your answers are important to us!

1.You are a: □ male □ female

2.Your age is ...... years old

3.In which department are you/were you hospitalized:...................................

4.Upon admission, were you accompanied from the admission service to the ward by:
□ a) healthcare personnel
□ b) family members (family members, friends)
□ c) you went alone
□ d) I don't know

5.Upon admission, were you informed about:
5.1. your rights and obligations as a patient? □ yes □ no
5.2. the rules and responsibilities during hospitalization? □ yes □ no

6.Were you satisfied with the quality of services provided during hospitalization?
6.1. accommodation □ a) yes □ b) no
6.2. bed quality, bedding □ a) yes □ b) no
6.3. cleanliness □ a) yes □ b) no
6.4. food quality □ a) yes □ b) no
6.5. variety of menus □ a) yes □ b) no
6.6. distribution and serving of meals □ a) yes □ b) no
6.7. medical support during internal transportation: □ yes □ no
6.8. time given for medical consultation □ a) yes □ b) no

7.Were you satisfied with the hospital environment during hospitalization?
7.1. appearance of the hospital/interior courtyard □ a) yes □ b) no
7.2. appearance of common spaces/department □ a) yes □ b) no
7.3. noise level □ a) yes □ b) no

8.Please evaluate the attitude, friendliness, availability, and quality of care provided by the following categories of personnel:
8.1. ward doctor: □ a) yes □ b) no
8.2. medical assistants: □ a) yes □ b) no
8.3. nurses: □ a) yes □ b) no
8.4. others (please specify).......... □ a) yes □ b) no

9.Were you satisfied with the care provided?
11.1 during the day □ a) yes □ b) no
11.2 during the night □ a) yes □ b) no
11.3 on Saturdays, Sundays, and legal holidays □ a) yes □ b) no
11.4 During internal transport □ a) yes □ b) no

10.The medications administered in the hospital during hospitalization:
□ a) were provided only by the hospital
□ b) were purchased by the family
□ c) both options

11.If the medications administered during hospitalization were purchased by the family, what was the procedure for acquisition?
□ a) on the simple prescription issued by the hospital doctor
□ b) on the prescription issued by the family doctor/specialist at the recommendation of the hospital doctor

12.Were you instructed on how to take oral medications (tablets, pills)?
□ a) yes
□ b) no

13.Were you instructed on how the intravenous treatment was administered?
□ a) yes
□ b) no

14.Were you informed of the identity of the medical staff involved in your treatment?
a) □ yes
b) □ no

15.Do you know of any adverse effects or risks associated with the medications or therapeutic procedures administered?
a) □ yes
b) □ no

16.Were you informed of the estimated date of discharge?
a) □ yes
b) □ no

17.Were you informed of the established diagnosis?
a) □ yes
b) □ no

18.Were you satisfied with the communication made by the hospital staff with you during your hospitalization?
□ a) yes
□ b) no

19.If it were necessary to be re-hospitalized, would you choose the same hospital/recommend our hospital to others?
□ a) definitely yes
□ b) probably yes
□ c) don't know
□ d) probably no
□ e) definitely no

20.Do you feel that your patient rights were respected?
□ a) yes
□ b) no
□ c) partially

21.How would you rate the quality of the recreational facilities and related activities offered throughout your hospitalization?

□ a) unsatisfactory
□ b) good

Observations and suggestions regarding the positive and/or negative aspects of the medical care during hospitalization:

Thank you for your cooperation!
