# Supplementary material for: The Impact of the COVID-19 Pandemic on the Management of Mental Health Services for Hospitalized Patients in Sibiu County—Central Region, Romania
Source: Healthcare (Basel). 2023 Apr 30;11(9):1291. doi: 10.3390/healthcare11091291 (PMC10178149; doi:10.3390/healthcare11091291)
Supplement: Supplementary file 1 [file healthcare-11-01291-s001.zip › Table S1.pdf]

SUPPLEMENTARY MATERIAL

Table S1 – Impact of the challenges illustrated by the study's results on the measures taken.

Challenges (based on results)	Measures taken
1. Decreased patient satisfaction	1. Individual approach<br>2. Distribution of resources necessary for recreation (TV/ INTERNET)<br>3. Establishment of a special place designed for the visit of patients' relatives according to the epidemiological norms.
2. Managing the increase in the number of admissions of psychiatric patients through the emergency department	1. Increasing material and human resources redirected to the emergency department.<br>2. Increasing the capacity of the emergency department (allocating supplementary space, supplies, and human resources)
3. Managing patients who avoided hospital medical care	1. Introduction of Telemedicine service in specialized outpatient clinics<br>2. Establishment of the telephone line TELVERDE
